# Low bone mineral density is a significant risk factor for low-energy distal radius fractures in middle-aged and elderly men: A case-control study

**DOI:** 10.1186/1471-2474-12-67

**Published:** 2011-04-02

**Authors:** Jannike Øyen, Gudrun Rohde, Marc Hochberg, Villy Johnsen, Glenn Haugeberg

**Affiliations:** 1 Department of Public Health and Primary Health Care, University of Bergen, Bergen, Norway; 2 Department of Rheumatology, Sørlandet Hospital, Kristiansand, Service box 416, 4604 Kristiansand, Norway; 3 Faculty of Health and Sport, University of Agder, Kristiansand, Norway; 4 Departments of Medicine and Epidemiology and Public Health, University of Maryland School of Medicine, Baltimore, Maryland, USA; 5 Department of Neuroscience, Division of Rheumatology, Norwegian University of Science and Technology, Trondheim, Norway

## Abstract

**Background:**

In general there is a lack of data on osteoporosis and fracture in men; this also includes low-energy distal radius fractures. The objectives of this study were to examine BMD and identify factors associated with distal radius fractures in male patients compared with controls recruited from the background population.

**Methods:**

In a 2-year period, 44 men 50 years or older were diagnosed with low-energy distal radius fractures, all recruited from one hospital. The 31 men who attended for osteoporosis assessment were age-matched with 35 controls. Demographic and clinical data were collected and BMD at femoral neck, total hip and spine L2-4 was assessed by dual energy X-ray absorptiometry.

**Results:**

Apart from weight and living alone, no significant differences were found between patient and controls for demographic variables (e.g. height, smoking) and clinical variables (e.g. co-morbidity, use of glucocorticoids, osteoporosis treatment, falls and previous history of fracture). However, BMD expressed as T-score was significant lower in patients than in controls at all measurement sites (femoral neck: -2.24 vs. -1.15, p < 0.001; Total hip: -1.65 vs. -0.64, p < 0.001; Spine L2-4: -1.26 vs. 0.25, p = 0.002). Among the potential risk factors for fracture evaluated, only reduced BMD was found to be significantly associated with increased risk for low-energy distal radius fractures in men.

**Conclusion:**

The results from our study indicate that reduced BMD is an important risk factor for low-energy distal radius fracture in men. This suggests that improvement of BMD by both pharmacological and non-pharmacological initiatives may be a strategy to reduce fracture risk in men.

## Background

The incidence of distal radius fractures is lower in middle-aged and elderly men than in women. In a Norwegian study, the average annual incidence rate was 25.4/10,000 in men compared with 109.8/10,000 in women over the age of 50 years [1].

Compared to women there is a general lack of data on distal radius fractures in men including data on prevalence of osteoporosis and risk factors [2-4]. In a few studies, osteoporosis has been reported to be more frequent in male distal radius fracture patients compared to control subjects and reference populations [2,5-7]. There is also a lack of data exploring the role of bone mineral density (BMD) together with other possible risk factors for low-energy distal radius fractures in men.

The objective of this prospective case-control study was to compare BMD at spine and hip in male low-energy distal radius fracture patients with age-matched controls and to search for factors associated with increased risk for distal radius fractures.

## Methods

### Study design and study population

The low-energy distal radius fracture patients (age ≥ 50 years) were all prospectively recruited from one hospital located in southern Norway in the two year period January 2004 through December 2005. The hospital is the only referral centre for orthopaedic trauma in the region. The population of men aged 50 years and older living in the recruitment area of the hospital is approximately 16,000 and the majority of the population lives in urban areas.

According to routine procedures at the hospital, all patients with fractures are identified by trained nurses and invited for osteoporosis assessment and fracture risk assessment at the osteoporosis centre. Patients with obvious confusion or dementia or serious infections were not invited for osteoporosis assessment. At the osteoporosis centre, routine clinical data were collected for fracture risk assessment, and BMD was measured (see below).

A low-energy distal radius fracture was defined as a fracture resulting from minimal trauma falling from standing height or less [8]. Patients with high-trauma fractures (e.g., occurring as the result of a motor vehicle accident) were excluded. A distal radius fracture was defined as located within 3 cm of the radio-carpal joint [9].

Age-matched controls were randomly identified in the national registry for the hospital catchment area and were invited by mail to participate in the study. We aimed to include one control person, matched for age and sex, for each patient. The controls were recruited and examined in the time period between June 2004 and March 2006.

The study was approved by the National Data Inspectorate and the regional committees for medical research ethics.

### Demographic and clinical data and BMD measurements

The demographic and clinical characteristics of the patients and controls are listed in table 1. The information was obtained partly by self-report questionnaire and partly by interview and clinical examination.

**Table 1:** Demographic variables, clinical characteristics and bone density measures in male distal radius fracture patients and controls

Demographics	Patients (n = 31)	Controls (n = 35)	P-value
Age (years)	66.5 (11.6)	66.2 (10.1)	0.916
Height (cm)	175.3 (9.7)	176.2 (5.2)	0.666
Weight (kg)	77.3 (14.5)	84.7 (10.7)	0.020
Education > 13 years	8 (32.0)	18 (51.4)	0.188
Living alone	12 (38.7)	3 (8.6)	0.007
Current smoker	10 (33.3)	8 (22.9)	0.411
Alcohol abuse	0 (0.0)	0 (0.0)	-
**Clinical characteristics**			
Rheumatoid arthritis	1 (3.2)	0 (0.0)	0.470
Inflammatory diseases	1 (3.2)	1 (2.9)	1.000
Endocrine diseases°	1 (3.2)	1 (2.9)	1.000
Cardiovascular diseases^	4 (12.9)	7 (20.0)	0.521
Diabetes	1 (3.2)	1 (2.9)	1.000
Glucocorticoids			
Ever	3 (11.5)	2 (5.9)	0.644
Current	2 (7.7)	0 (0.0)	0.184
≥ 3 months	2 (6.5)	2 (5.7)	1.000
Calcium supplement	3 (9.7)	3 (8.6)	1.000
Vitamin-D supplement	12 (38.7)	14 (40.0)	1.000
Bisphosphonate	2 (6.4)	1 (2.9)	0.597
Loss of height (≥ 3 cm)•	6 (21.4)	6 (17.6)	0.755
Previous fracture†	4 (12.9)	4 (12.9)	1.000
History of hip fracture in a parent	3 (10.3)	3 (9.1)	1.000
Falls (≥ 1 fall last year)	9 (40.9)	5 (18.5)	0.116
**Bone mineral density**			
BMD femoral neck (g/cm^2^)	0.78 (0.14)	0.92 (0.14)	<0.001
BMD total hip (g/cm^2^)	0.84 (0.16)	1.01 (0.13)	<0.001
BMD L2-4 (g/cm^2^)	1.08 (0.24)	1.27 (0.20)	0.001
T-score femoral neck (SD)	-2.24 (1.05)	-1.15 (1.11)	<0.001
T-score total hip (SD)	-1.65 (1.04)	-0.64 (0.91)	<0.001
T-score L2-4 (SD)	-1.26 (2.05)	0.25 (1.69)	0.002
Femoral neck			0.006**
Normal BMD	2 (6.9)	14 (40.0)	
Osteopenia	20 (69.0)	18 (51.4)	
Osteoporosis	7 (24.1)	3 (8.6)	
Total hip			<0.001**
Normal BMD	6 (23.1)	23 (65.7)	
Osteopenia	15 (57.7)	12 (34.3)	
Osteoporosis	7 (19.2)	0 (0.0)	
Lumbar spine L2-4			0.002**
Normal BMD	11 (35.5)	26 (74.3)	
Osteopenia	12 (38.7)	8 (22.9)	
Osteoporosis	8 (25.8)	1 (2.9)	
Normal BMD at all sites*	2 (6.5)	14 (40.0)	0.002
Osteopenia at ≥1 site but no osteoporosis*	20 (64.5)	17 (48.6)	0.222
Osteoporosis at ≥1 site*	9 (29.0)	5 (11.4)	0.120

Mean (SD) for continuous variables and numbers (%) for categorical variables.

SD: standard deviation. BMD: bone mineral density. Total numbers may vary between different variables according to different numbers of missing data. °Endocrine diseases included hyperparathyroidism, hypothyroidism and hyperthyroidism. ^Cardiovascular disease comprised angina pectoris and cardiac infarction. •Difference between present height and the given maximum adult height. †Previous fracture before the current distal radius fracture defined as a fracture of the distal radius, upper arm, rib, spine, hip, femur or lower leg from low-energy trauma after the age of 50. *Femoral neck, total hip, lumbar spine L2-4. **Overall p-value for the actual categorised variable.

BMD was measured at the femoral neck, total hip and lumbar spine (L2-4), by four trained nurses using the same dual energy X-ray absorptiometry (DXA) equipment (General Electric, Lunar Prodigy). The in-vitro long term coefficient of variance (CV) for the spine phantom was 0.62% for the entire study period. The in-vivo CV for the measurement procedure was 1.68% for right femoral neck, 1.56% for left femoral neck, 0.94% for the right total hip, 0.88% for the left total hip and 1.26% for lumbar spine L2-4. We used the BMD values for the left hip unless there was a history of previous fracture or surgery. Scans from the right hip were used in three patients.

T-score calculations were derived from standardized NHANES male reference population supplied by the DXA manufacturer. We used the WHO cut point definition for osteoporosis (T-score ≤ -2.5 standard deviation (SD)), osteopenia (T-score > -2.5 SD and < -1.0 SD) and normal bone density (T-score ≥ -1.0 SD) (10).

### Fracture risk estimated by FRAX^®^

We used the WHO fracture risk assessment tool FRAX^® ^to investigate if the distal radius fracture patients had higher fracture risk than the controls prior to their current distal radius fracture. In the absence of a Norwegian FRAX^® ^model, we used the Swedish FRAX^® ^model to estimate the 10-year risk of hip fractures and any major osteoporotic fractures (clinical spine, forearm, hip, or shoulder). FRAX^® ^was calculated with and without femoral neck BMD (g/cm^2^) in the algorithm. The clinical risk factors included in the FRAX^® ^model comprise information on race, age, sex, weight, height, femoral neck BMD, a previous fracture, parental history of hip fracture, current smoking, use of oral glucocorticoids for more than three months, rheumatoid arthritis, other secondary causes of osteoporosis, and alcohol intake of three or more units per day [11]. The FRAX^® ^was calculated without taking the current distal radius fractures into account, but earlier low-energy fractures were registered.

### Statistical analysis

Categorical variables were expressed as numbers and percentages, and continuous variables as means with SD or as medians with range. We used independent-samples t-test for continuous variables and chi-square test for categorical variables in comparisons between fracture patients and controls, and between distal radius fracture patients attending and not attending for DXA. Chi-square tests were used to explore for differences in frequency of patients with distal radius fractures in spring, summer, autumn and winter. Independent-samples t-test was also used to compare FRAX^® ^10-year probability for fracture between fracture patients and controls.

The association between low-energy distal radius fractures as dependent variable and the variables included in table 1 as independent variables were tested in conditional logistical regression analyses, unadjusted and adjusted for age. The degree of association was expressed as odds ratio (OR) with 95% confidence interval (CI). A two-tailed p-value < 0.05 was considered statistically significant. All analyses were performed using SPSS software for Windows, version 15.0 (SPSS Inc., Chicago, Illinois).

## Results

In the two-year period, a total of 44 men with low-energy distal radius fractures resident in the geographic area of the hospital were assessed and treated. Two male tourists with low-energy distal radius fracture were also treated at the hospital but excluded from the present analysis. Among the 44 resident patients, two were excluded due to dementia or confusion, and 11 patients were unwilling to be assessed at the osteoporosis centre. The final study sample comprised 31 patients, giving a response rate among potentially eligible patients of 67.4%. For the age groups 50-59 years (n = 11), 60-69 years (n = 7) and 70 and above (n = 13) the response rate was 84.6%, 63.6% and 65.0%, respectively. The median time between fracture and examination at the osteoporosis centre was 10.0 days (range 1-50 days).

Resident patients not assessed at the osteoporosis centre were on average 4.6 years older than patients assessed at the osteoporosis centre (mean 71.1 years, SD 10.7 vs. 66.5 years, SD 11.6; p = 0.10).

A total of 65 potential control subjects were invited to participate, of which 35 were willing to participate, giving a response rate of 54%.

### Distal radius fracture prevalence and seasonal variations

No statistically significant difference in the distribution across seasons was seen among the male distal radius fracture patients; however, numerically more patients had a fracture in winter (n = 16) than in spring (n = 11), summer (n = 7) and autumn (n = 10). There also was no significant difference in the proportion of patients with distal radius fractures with osteoporosis between the seasons (spring 16.7%, summer 40.0%, autumn 25.0% and winter 33.3%).

### Distal radius fracture patients and age-matched controls

Demographic variables, clinical characteristics and BMD values for patients assessed at the osteoporosis centre and controls are shown in table 1. The patients had significant lower weight than the control subjects, and more patients than controls were living alone. The patients had significant lower BMD and T-scores than the controls at all measurements sites. More patients also had osteoporosis and osteopenia than controls. For the other demographic and clinical variables listed in table 1, no significant differences were seen between the two groups.

### Risk factors for distal radius fractures

ORs for the association of low BMD with distal radius fractures were estimated in unadjusted and age-adjusted analyses. As shown in table 2, both osteoporosis and osteopenia were significantly associated with distal radius fractures. Weight was found to be of borderline significance in both unadjusted (OR = 0.95, 95% CI: 0.91-1.00, p = 0.053) and age-adjusted models (OR = 0.96, 95% CI: 0.91-1.00, p = 0.064). Living alone was no longer significantly associated with fracture in unadjusted or age-adjusted models (data not shown).

**Table 2:** Unadjusted and age adjusted odds ratios (OR*) with 95% confidence interval (CI) for associations between osteopenia and osteoporosis and low-energy distal radius fracture in men

	Unadjusted		Adjusted for age	
		
	OR (95%) CI	p-value	OR (95%) CI	p-value
Osteopenia †	5.95 (1.32-26.86)	0.020	5.82 (1.28-26.40)	0.023
Osteoporosis‡	10.48 (1.63-67.60)	0.013	10.02 (1.54-65.32)	0.016

* OR and 95% CI was calculated using conditional logistic regression analysis. † At femoral neck, total hip and/or lumbar spine L2-4 but no osteoporosis. ‡ At femoral neck, total hip and/or lumbar spine L2-4.

When FRAX^® ^was calculated using femoral neck BMD, a significant difference in 10-year risk for hip fracture (9.6% vs. 4.7%, p = 0.039) and for any major osteoporotic fracture (15.5% vs. 10.0%, p = 0.033) was found between distal radius fracture patients and controls. However, when FRAX^® ^was calculated using weight and height but without BMD, the differences in FRAX^® ^between the two groups was no longer significant (hip fracture: 5.0% vs. 4.0%, p = 0.53; any major osteoporotic fracture: 10.0% vs. 8.8%, p = 0.51).

## Discussion

The most striking result from the present case-control study is the demonstration of an approximately 15% lower BMD at the hip and spine in men with distal radius fractures compared with age-matched controls and the apparently strength of the association between low BMD and low-energy distal radius fracture in men. This study adds evidence that reduced BMD is a major risk factor for low-energy distal radius fracture in middle-aged and elderly men as previously has been shown for women [12-14]. In the present study, no other risk factors were found to be significantly associated with low-energy distal radius fractures, although there was a trend for low body weight. This most likely is related to the low number of patients included in our study.

The strengths of this study are the prospective study design for case acquisition over a 2-year period, high participant rate (67.4% of all cases identified within a single geographic region), the short time between the occurrence of the fractures and the examination at the osteoporosis centre (median 10 days) and use of age-matched controls recruited randomly from the population. We also identified all male patients with low-energy distal radius fracture including patients not attending DXA-measurements at the osteoporosis centre. Further, we only included patients defined according to the definition of low-energy fractures and not high-energy trauma patients.

However, a limitation of this study is the rather low number of fracture patients included. This may have limited our ability to identify other important risk factors than reduced BMD for low-energy distal radius fracture in men. Thus, our results should be interpreted with caution. Furthermore, the response rate among controls who were invited for assessment was only 54%. This may have biased our results as the control group may not reflect the true background population regarding distribution of risk factors for distal radius fracture.

In our study more male fracture patients than male controls were living alone. In a recently published case-control study examining 214 female low-energy distal radius fracture patients recruited from the same geographic area over the same two year period, we found that living alone and current use of glucocorticoids in addition to having osteoporosis were independently associated with low-energy distal radius fracture in women [14]. The explanation for this may be that behavioral and psychological factors associated with living alone, might influence the risk of falls and fractures [15].

The discrepancy in numbers between men and women in these two studies from the same geographic area is reflected by the different age specific fracture rates of approximately 1:4 reported for individuals aged ≥ 50 years with distal radius fracture in Norway [1].

Environmental factors may also contribute to an increased risk of distal radius fracture. Previous studies have shown that distal radius fractures occur more frequently in the winter months in both men and women [4,16,17] and especially on days with snow and ice [18]; this probably is due to an increased risk of falling. In our study, we also found a tendency to an increased risk of distal radius fracture in men in winter as we previously have also shown for women [14].

Our study confirms the results from previous cross-sectional studies which have reported DXA BMD at heel, spine and hip to be lower in male distal radius fracture patients than controls [2,6]. In the study by Tuck et al that including men with high-energy distal radius fracture, the magnitude of the difference in BMD at lumbar spine (7%), total hip (8%) and femoral neck (12%) was lower compared with that seen in our study (~15% both at spine and hip) [2]. In that study, the authors also reported that osteoporosis was independently associated with increased risk of distal radius fracture after adjusting for age and body mass index (BMI).

Among studies in men with distal radius fracture without bone density data, one retrospective case-control study reported a history of previous fracture to be the only independent risk factor for low-energy fracture [19], whereas another prospective study reported increasing age and reduced BMI to be predictors of increased forearm fracture risk [20]. In our study, mean weight was significantly lower in men with distal radius fracture; however, this was less strongly associated with distal radius fracture than reduced BMD. Other tested potential risk factors in our study including falls during the past year and previous low-energy fractures were also not found to be statistical significantly associated with an increased fracture risk. This again is most likely due to the low number of patients included as in our study.

In low-energy distal radius fracture patients, reduced BMD has not only been shown to be related to an increased fracture risk but also to be associated with an increased severity of the distal radius fracture [21]. The importance of reduced BMD as a risk factor for our low-energy distal radius fracture patients is also demonstrated by the FRAX^® ^fracture risk analysis. We only found a statistically significant difference in FRAX^® ^between patients and controls when femoral neck BMD was included in the FRAX^® ^algorithm but not when the FRAX^® ^algorithm was calculated without BMD.

The results from the present study suggest that more focus should be placed on improving bone strength and reducing the risk of falling especially in winter time to reduce low-energy distal radius fracture risk in men above 50 years. Strategies to improve bone strength may include changes in life style factors (e.g. modifying drinking and smoking habits), improving nutrition including calcium and vitamin D supplementation, and medical treatment if indicated.

## Conclusions

The results from the present study indicate that low bone mineral density is an important modifiable risk factor for low-energy distal radius fracture in men. This suggests that strategies to improve bone strength should include both pharmacological and non-pharmacological initiatives. However, larger studies are needed to confirm our results as our study is limited by the low number of patients.

## Competing interests

The authors declare that they have no competing interests.

## Authors' contributions

JØ contributed substantially to analyse and interpret the data and was involved in drafting the manuscript. GR participated in acquisition of data, interpretation of data and revising the manuscript critically for important intellectual content. MH and VJ contributed substantially to the conception and study design, interpreting the data and revising the manuscript critically for important intellectual content. GH was the principal investigator and he was involved in all steps of the study including conception of the study, design of the study, acquisition of data, data analysing and interpretation of data and drafting manuscript. All authors have given final approval of the version to be published.

## Pre-publication history

The pre-publication history for this paper can be accessed here:

http://www.biomedcentral.com/1471-2474/12/67/prepub
